# Role of SH3GLB1 in the regulation of CD133 expression in GBM cells

**DOI:** 10.1186/s12885-023-11211-8

**Published:** 2023-07-31

**Authors:** Chia-Hung Chien, Chien-Cheng Lai, Jian-Ying Chuang, Jui-Mei Chu, Chan-Chuan Liu, Kwang-Yu Chang

**Affiliations:** 1 National Institute of Cancer Research, National Health Research Institutes, 367 Sheng-Li Road, Tainan, 70456 Taiwan; 2 School of Medicine, I-Shou University, Kaohsiung, Taiwan; 3 International Master Program in Medical Neuroscience, Taipei Medical University, Taipei, Taiwan; 4 Department of Biomedical Science and Environmental Biology, Kaohsiung Medical University, Kaohsiung, Taiwan; 5 Department of Oncology, National Cheng Kung University Hospital, College of Medicine, National Cheng Kung University, Tainan, Taiwan

**Keywords:** SH3GLB, Resistance, TICs, CD133, Histone acetylation

## Abstract

**Background:**

Glioblastoma (GBM), a malignant brain tumor, has poor survival outcomes due to recurrence or drug resistance. We found that SH3GLB1 is a crucial factor for cells to evade temozolomide (TMZ) cytotoxicity through autophagy-mediated oxidative phosphorylation, which is associated with CD133 levels. Therefore, we propose that SH3GLB1 participate in the impact on tumor-initiating cells (TICs).

**Methods:**

The parental, the derived resistant cell lines and their CD133^+^ cells were used, and the levels of the proteins were compared by western blotting. Then RNA interference was applied to observe the effects of the target protein on TIC-related features. Finally, in vitro transcription assays were used to validate the association between SH3GLB1 and CD133.

**Results:**

The CD133^+^ cells from resistant cells with enhanced SH3GLB1 levels more easily survived cytotoxic treatment than those from the parental cells. Inhibition of SH3GLB1 attenuated frequency and size of spheroid formation, and the levels of CD133 and histone 4 lysine 5 (H4K5) acetylation can be simultaneously regulated by SH3GLB1 modification. The H4K5 acetylation of the CD133 promoter was later suggested to be the mediating mechanism of SH3GLB1.

**Conclusions:**

These data indicate that SH3GLB1 can regulate CD133 expression, suggesting that the protein plays a crucial role in TICs. Our findings on the effects of SH3GLB1 on the cells will help explain tumor resistance formation.

**Supplementary Information:**

The online version contains supplementary material available at 10.1186/s12885-023-11211-8.

## Background

Glioblastoma (GBM) is a fatal disease with bad outcomes, and the prognosis has not improved even with advanced treatment. Despite the outcomes, a first-line chemotherapeutic drug, temozolomide (TMZ), is considered to control disease by inducing lethal DNA damage [1]. However, most patients experience tumor recurrence [2], and the mechanism of TMZ resistance is complicated as multiple factors are involved in it. Therefore, understanding its mechanism would significantly contribute to the therapeutic benefits. The cells express O6-methylguanine-DNA methyltransferase (MGMT), allowing them to escape drug cytotoxicity [3].

Apart from MGMT leading to TMZ resistance, tumor-initiating cells (TICs) in gliomas may be involved in the resistance. Studies indicate that TICs are responsible for drug-acquired resistance [4]. This specific subgroup of cells is characterized by self-renewal and multipotency, referred to as stem cell properties [5]. Accumulating studies have demonstrated that CD133 is a valuable TICs marker to predict the recurrence of high-grade glioma [6–8]. Our previous study showed that long-term treatment with TMZ caused the acquired resistance derived from the enrichment of the TIC’s properties by increasing superoxide dismutase 2 (SOD2) accompanied with CD133 up-regulation [9]. Suberoylanilide hydroxamic acid, a histone deacetylases inhibitor, reduced the levels of stemness-related markers and viability of glioma stemness-featured cells while inducing apoptosis and senescence, which can reduce resistance [10].

SH3GLB1, also called Bax interacting factor 1 (Bif-1) or Endophilin B1, belongs to the endophilin family and contains an N-terminal BAR domain and a C-terminal SH3 domain implicated in signal pathways and the activity of membrane reshaping [11]. The membrane curvature function of SH3GLB1 is involved in mitochondrial dynamics, autophagy, apoptosis, and endocytosis. In the previous studies, we found that SH3GLB1 was increased in GBM-resistant cells, and the protein can mediate oxidative phosphorylation, which leads to TMZ-induced acquired resistance through autophagy. Moreover, SH3GLB1 was involved with mitochondrial functions and stemness features, especially at CD133 levels [12]. CD133 expression was associated with histone H4 acetylation [13], and the enhanced mitochondrial activity was related to histone H4 lysine 5 (H4K5) acetylation [14]. Therefore, we further investigated whether there was an association between SH3GLB1 and CD133.

## Materials and methods

### Cell culture

The human GBM cell lines, U87MG and A172, were purchased from the American Type Culture Collection (ATCC, VA, USA). Patient #5 cells were derived from a patient with GBM, as described in the previous studies [12]. They were cultured in a medium containing Dulbecco’s modified Eagle’s medium (DMEM) (Thermo Fisher Scientific, NMA, USA) with 10% fetal bovine serum (FBS) and 1–2% penicillin/streptomycin (Thermo Fisher Scientific). The resistant cell lines (U87MG-R and A172-R) were constructed from the parental U87MG and A172 cells in our previous study. The maintenance of the cells was performed similarly to that of the parental cells [9, 15, 16]. The cells transfected with a lentiviral GFP plasmid (System Biosciences, CA, USA) were used as a tracer in a co-culture experiment. GFP-carrying cells were enriched using cell sorting during flow cytometry (FACSAria™ III, BD Biosciences, CA, USA) analysis. A primary GBM recurrent tumor, P1S [17], was obtained from a surgical sample of a patient undergoing multiple treatments and developing drug resistance. The tumor was maintained in NOD-SCID mice.

### Sorting of CD133^+^ cells

Fluorochrome-conjugated anti-CD133 antibodies (Miltenyi Biotec, Bergisch Gladbach, Germany) were attached to the cells and sorted using FACSAria™ III (BD Biosciences) flow cytometry. The enriched CD133^+^ cells were cultured in DMEM/F12 (Thermo Fisher Scientific) serum-free medium supplemented with 2% B27 (Thermo Fisher Scientific), 10 ng/mL epidermal growth factor (ProSpec, NJ, USA), and 10 ng/mL fibroblast growth factor 2 (Cell Guidance Systems, Cambridge, United Kingdom).

### Chemical reagents and antibodies

TMZ and H_2_O_2_ were purchased from Sigma-Aldrich (MO, USA). The following antibodies were used for western blotting and chromatin immunoprecipitation. SH3GLB1 and CD133 were purchased from Proteintech (IL, USA). Caspase 3 was purchased from Cell Signaling (MA, USA). Acetyl H4K5 antibody for chromatin immunoprecipitation and actin antibodies were purchased from Merck Millipore (MA, USA). Acetyl H4K5 antibody for western blotting was purchased from GeneTex, (CA, USA).

### Tumor spheroid formation assays

We used Extreme Limiting Dilution Analysis (ELDA) to evaluate self-renewal functions in GBM TICs. The cells in DMEM/F12 medium with serum-free growth factors and 0.3% methylcellulose (Sigma–Aldrich) in ultra-low adherent plates were seeded at densities of 1, 5, 10, 20, and 50 cells per well in suspension culture, as described in our previous studies [9]. After 2 weeks, the number of spheres was measured, and the frequency of cell initiation was calculated using web software (http://bioinf.wehi.edu.au/software/elda/).

### Fluorescent staining

MitoTracker Red CMXRos (Thermo Fisher Scientific) and DAPI (Thermo Fisher Scientific) were used to detect the mitochondrial location and nucleus position, respectively. The goat anti-rabbit secondary antibody (Alexa Fluor™ 488, Thermo Fisher Scientific) was used to target the SH3GLB1 primary antibody. The staining results were detected using a fluorescence microscope (Nikon TE200, Tokyo, Japan).

### RNA-based gene modulation of SH3GLB1

In transient transfection, we used Lipofectamine® RNAiMAX and LTX with Plus™ reagents (Thermo Fisher Scientific) mixing with SH3GLB1 siRNA (GenePharma, Shanghai, China) or an SH3GLB1 vector (GenScript Biotech, NJ, USA) to examine short-term gene expression. In stable gene knockdown, SH3GLB1-lentiviral short hairpin RNA (shRNA) or an empty vector (both from RNAi Core, Academia Sinica, Taiwan) were used to infect the cells. The stable clones were selected in the medium with an antibiotic for weeks.

### Western blotting

The indicated proteins were prepared from cell or tissue lysates. After quantification, we performed sodium dodecyl sulfate-polyacrylamide gel electrophoresis (SDS-PAGE) and transferred the bands to polyvinylidene difluoride (PVDF) membranes (Bio-Rad, CA, USA). Since the positions where all protein blots appeared were quite stable and for obtaining clearer western blot bands, we set the upper and lower boundaries of the membranes according to protein molecular weight, and the left and right boundaries were according to different cell lines or other experiments. Therefore, all the blots were cropped prior to hybridization with primary antibodies. We used 5% non-fat milk to block membranes from non-specific binding. We incubated the blot with primary antibodies at 4 °C. After tagging with secondary antibodies, the immunoblot signals and intensity were detected using ECL (enhanced chemiluminescence) substrates.

### Chromatin immunoprecipitation (ChIP)

The ChIP assay was conducted following our published methodology [12]. Normal human IgG (GeneTex, CA, USA) was used as a negative control. Subsequent analysis in qPCR was done with CD133 primers, as follows: Forward 5’-CCGGCA GTGGGAGGCGGGCT-3’ and Reverse 5’-CACCCCCAGTACAGTGGAAG-3’. The total RNA isolation and quantification were performed similarly to the previous studies [12]. The percentage of immunoprecipitated chromatin vs. total chromatin (input) was calculated for normalization.

### Reporter assay

We used the Nano-Glo® Dual-Luciferase® Reporter Assay System (Promega, WI, USA) to measure luminescent signals according to the manufacturer’s instructions. The cells were co-transfected with the NanoLuc® Luciferase (Nluc) Reporter Vector (pNL1.1[Nluc] Vector) and the Firefly Luciferase (Fluc) Reporter Vector (pGL4.54 [luc2/TK] Vector). The Fluc vector was used to normalize transfection efficiency. The results were represented as the ratio of the Nluc activity to the Fluc activity ([Nluc]/[luc2/TK]) calculated as relative luciferase activity.

### Summary Graph

We used online software, BioRender (http://biorender.io), to develop our schematic diagram.

### Statistics

The results were statistically analyzed using Prism (CA, USA). A two-tailed Student’s t-test for two samples was used to calculate whether the two groups differed. The difference was considered significant, if p < 0.05.

## Results

### The resistant CD133^+^ cells with increased SH3GLB1 levels at the top of the hierarchy escape cytotoxicity

**Fig. 1:**
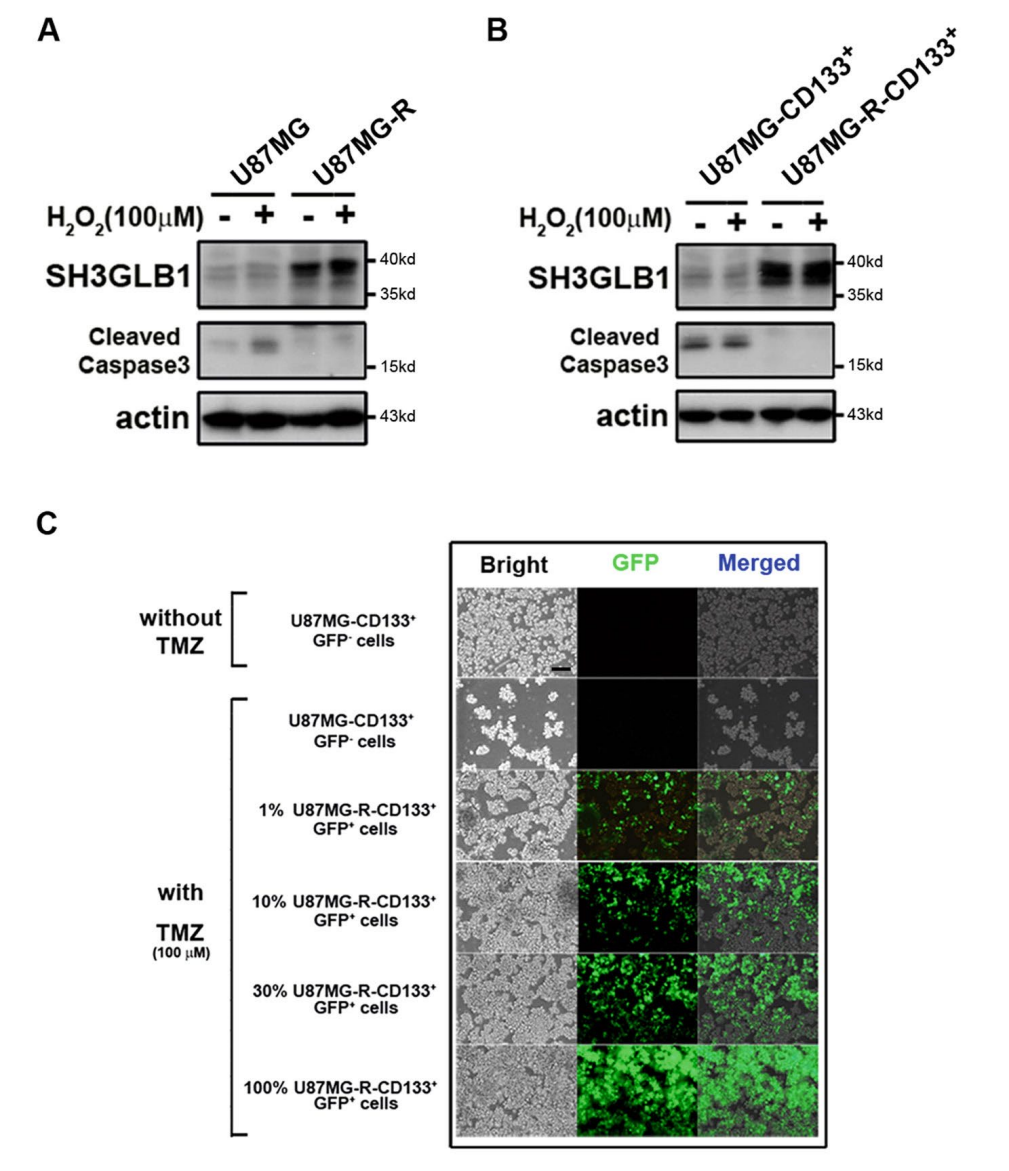
High SH3GLB1 contributed to specialized cell-fate decisions. (**A**) U87MG parental (U87MG) and the TMZ-resistance cells (U87MG-R), and (**B**) the sorted CD133^+^ subsets from the U87MG and U87MG-R. The resistant cells were associated with the cells withstanding external H_2_O_2_ (100 µM; 24 h) stress. (**C**) The resistant CD133^+^ cells carrying GFP expression were co-cultured in the indicated percentage with the parental cells without GFP labeling following TMZ (100 µM) treatment for 24 h. GFP: Green fluorescent protein; TMZ: temozolomide. All the blots were cropped prior to hybridization with primary antibodies. The original blots are presented in Fig. S3

Our previous studies showed that resistant CD133^+^ cells with SH3GLB1 deficiency could promote cell death after TMZ treatment [12]. However, the roles of SH3GLB1 on TICs or hierarchical cells are still unclear. To realize the significance, hydrogen peroxide (H_2_O_2_), a mediator that efficiently causes cell death [18], was applied. The results showed that the parental cells (Fig. 1A) or the sorted CD133^+^ cells (Fig. 1B) from the parental cells had lower levels of SH3GLB1, making the cells sensitive to H_2_O_2_. However, the resistant cells, or the CD133^+^ cells from the resistant cells carrying higher SH3GLB1 levels, easily survive the treatment. In contrast, different percentages of resistant CD133^+^ cells were co-cultured with the parental cells to examine the different cellular hierarchies. As shown in Fig. 1C, the resistant CD133^+^ cells contributed to the main survivors in the TMZ treatment but not the parental CD133^+^ cells. These results suggested that SH3GLB1 can decide the fate of the specific cell groups after the cytotoxic regimen.

### SH3GLB1 contributed to the formation of tumor-initiating cells

**Fig. 2:**
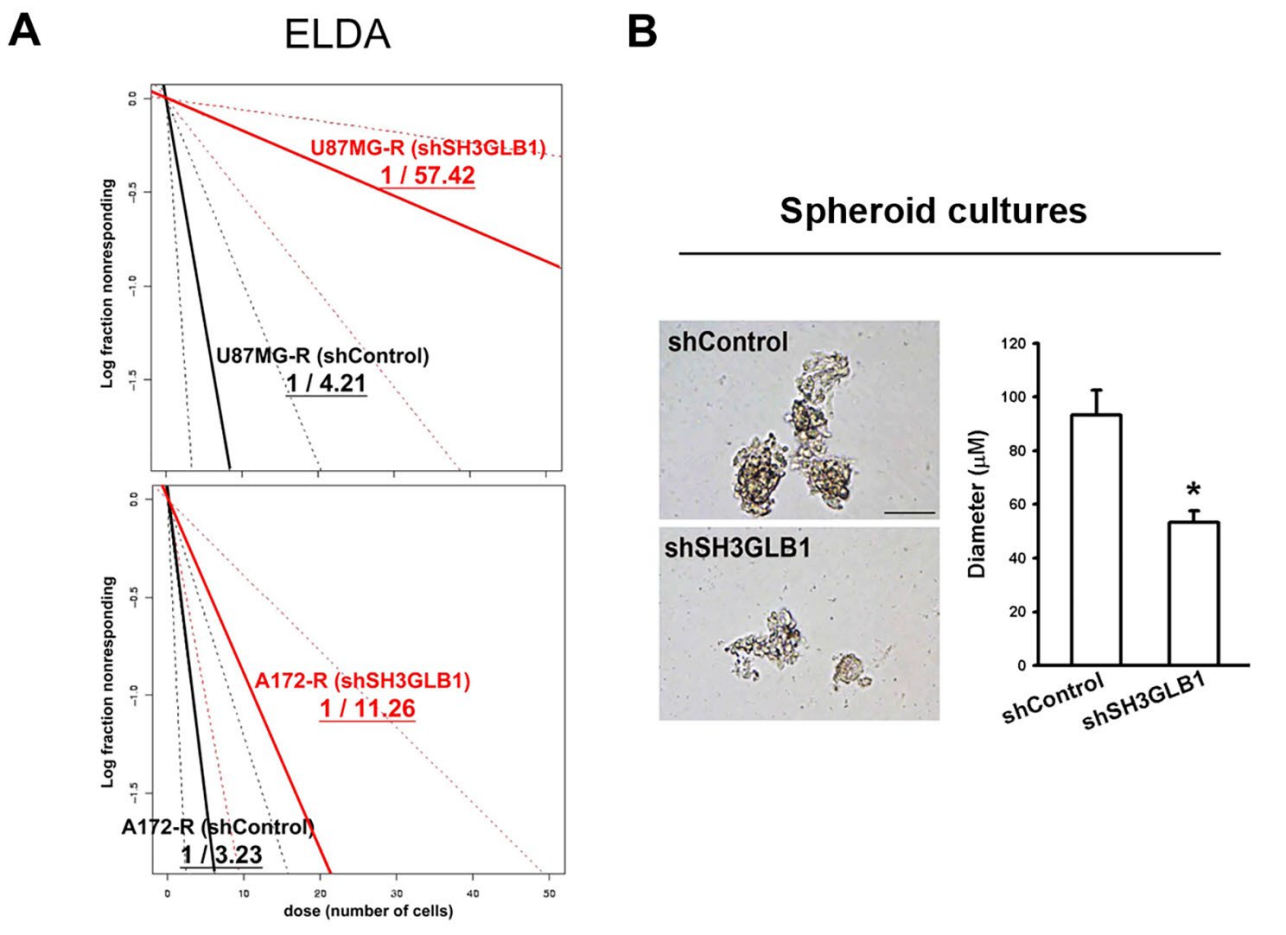
SH3GLB1 affects TIC features. (**A**) The frequency of the spheroid formation in U87MG-R (upper panel) and A172-R (lower panel) cells was estimated using an in vitro extreme limiting dilution assay. (**B**) The sphere formation was observed using SH3GLB1 knockdown in primary resistant cells (P1S). The statistical diameters are shown, and the scale bar is 100 μm. *p < 0.05. TIC: tumor-initiating cell

We further examined the impact of SH3GLB1 on the TIC features. Using an in vitro ELDA study, we showed that the stemness frequency decreased from 1/4.21 to 1/57.42 in U87MG-R cells (Fig. 2A, upper) and from 1/3.23 to 1/11.26 in A172-R cells (Fig. 2A, lower) following SH3GLB1 knockdown. In addition, the data demonstrated that the loss of SH3GLB1 in primary TMZ-R cells disturbed tumor sphere formation (Fig. 2B). These results suggested that SH3GLB1 can impact the development of the tumor-initiating cells.

### SH3GLB1 affects CD133 expression via histone H4K5 acetylation

**Fig. 3:**
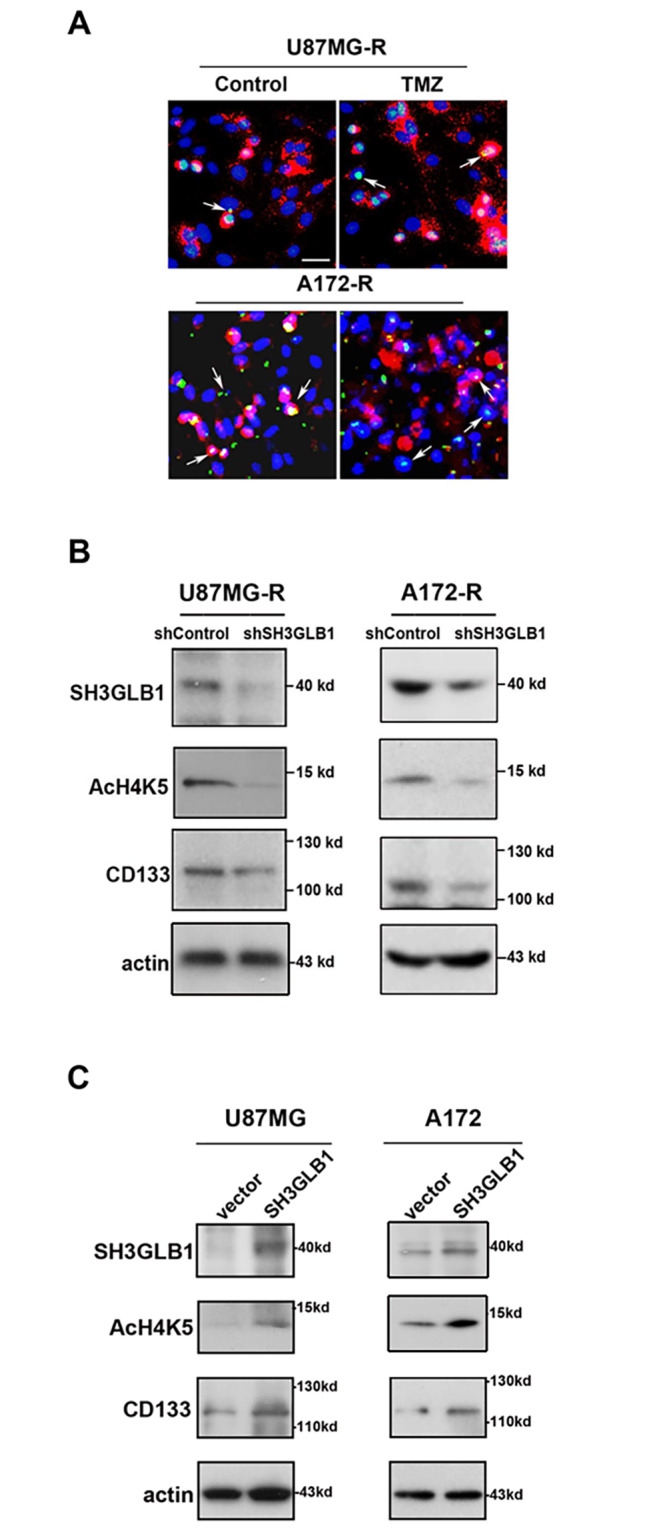
SH3GLB1 regulates CD133 expression. (**A**) Immunofluorescence staining shows the distribution of SH3GLB1 in the resistant cells. Green: SH3GLB1; red: MitoTracker Red CMXRos; blue: DAPI; scale bar = 250 μm SH3GLB1 shRNA or overexpression vector was used in U87MG- and A172-resistant cells (**B**) or parental cells (**C**), respectively. The association between SH3GLB1, acetylated histone H4 lysine 5 (AcH4K5) and CD133 was studied using western blotting. All the blots were cropped prior to hybridization with primary antibodies. The original blots are presented in Fig. S4 and S5

Using immunofluorescence, SH3GLB1 was found to be distributed in the nucleus, cytoplasm, and mitochondria (Fig. 3A). Since SH3GLB1 can be expressed in the nucleus, we wanted to examine whether it was involved in gene regulation. Supportively, clinical database showed that there was a positive correlation between the levels of SH3GLB1 and CD133 in GBM (Fig. S1). As shown in Fig. 3B, when SH3GLB1 was down-regulated in the resistant cells, the histone H4K5 acetylation and CD133 were reduced. In contrast, SH3GLB1 overexpression resulted in increased levels of histone H4K5 acetylation and CD133 in the parental cells (Fig. 3C). These results suggest SH3GLB1 can regulate CD133 expression through histone H4K5 acetylation.

**Fig. 4:**
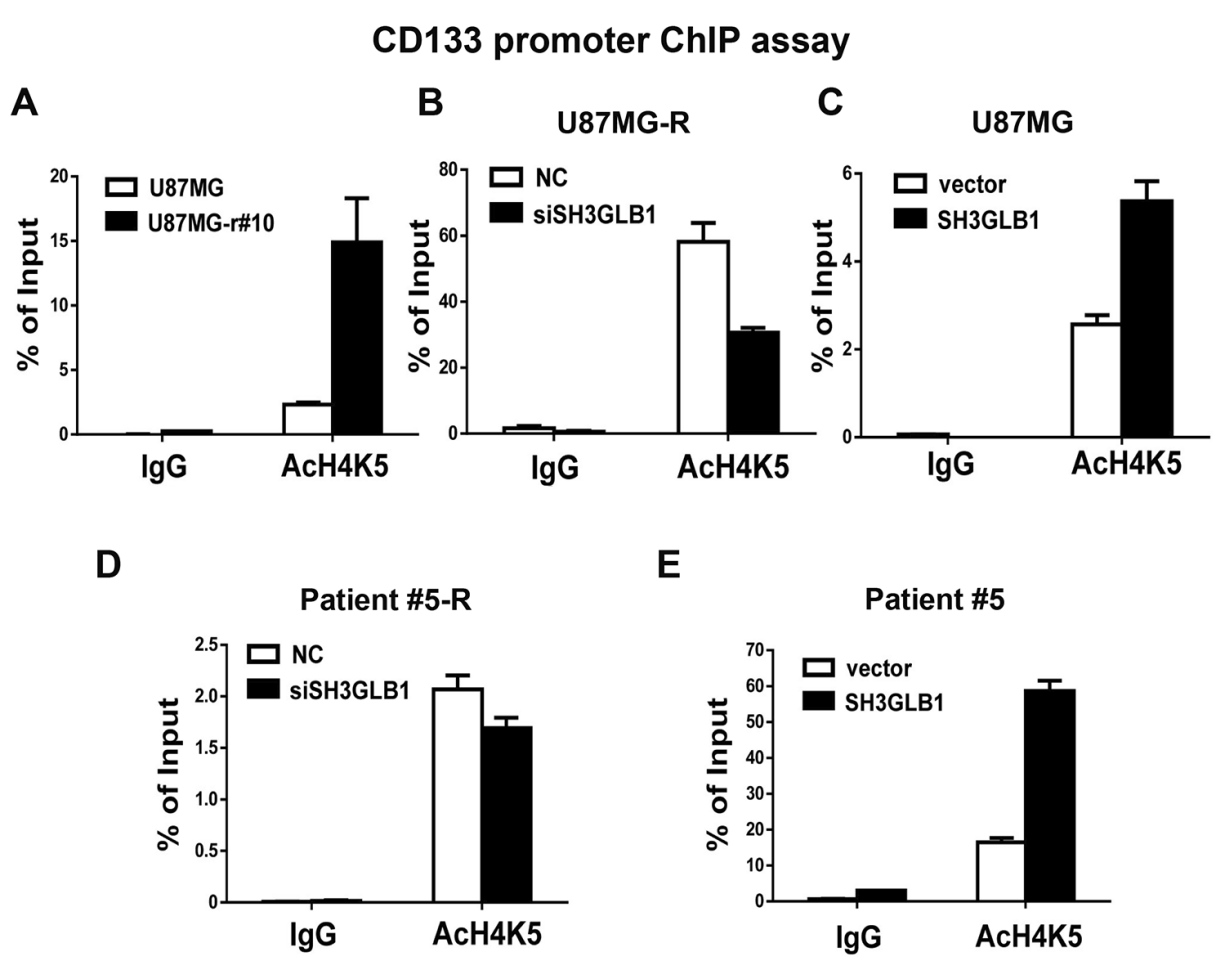
In chromatin immunoprecipitation assay, analysis of SH3GLB1 to histone H4K5 acetylation on the CD133 promoter by transfecting SH3GLB1 siRNA or overexpression vector in resistant (U87MG-R and Patient #5-R) or paired parental (U87MG and Patient #5) cells. The groups are indicated by different bar graphs (white or black). R: resistant

**Fig. 5:**
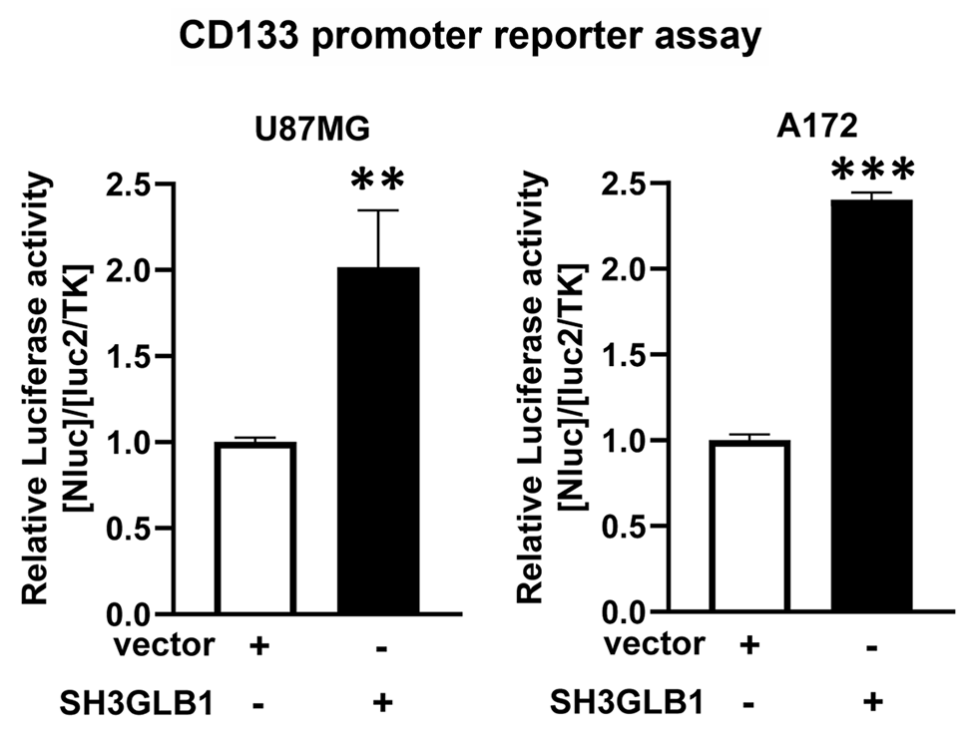
Luciferase reporter assays showed that the CD133 promoter activity was enhanced by transfecting SH3GLB1 overexpression vector in U87MG or A172 parental cells. **p < 0.01; ***p < 0.001

We examined the association between SH3GLB1 and histone H4K5 acetylation using a ChIP assay. As shown in Fig. 4A, histone H4K5 acetylation was found in the CD133 promoter region, and the acetylation levels were significantly enhanced in the resistant cells compared to the parental cells. Moreover, no matter whether in resistant or parental cells derived from cell lines (Fig. 4B, C) and primary clinical cells (Fig. 4D, E, Fig. S2A), it was found SH3GLB1 down-regulation in the resistant cells reduced histone H4K5 acetylation on CD133 promoter and SH3GLB1 overexpression in the parental cells enhanced the acetylation on CD133 promoter. The luciferase reporter assay was performed to examine the activity of the CD133 promoter with or without SH3GLB1 overexpression. The results showed that luciferase activity was enhanced as the cells were with SH3GLB1 up-regulation (Fig. 5). The results indicated that SH3GLB1 could regulate CD133 gene expression via acetylation of the histone H4K5.

## Discussion

TICs remained controversial in the development of tumor resistance. However, the fact that CD133^+^ cells are related to these ominous features within the tumor remained suggestive of their role [19]. In the previous study [12], we discovered that SH3GLB1 regulates on oxidative phosphorylation (OXPHOS) in the GBM cells, leading to TMZ resistance. We performed bioinformatic analysis on GBM cells to find a sub-population with enhanced SH3GLB1 and OXPHOS levels that had up-regulated CD133 expression. Here we further identified the important roles of SH3GLB1 on TIC features, spheroid growth (Figs. 1 and 2), and CD133 expression (Figs. 3, 4 and 5). It was found that TMZ-resistance cells expressed increased levels of SOD2 [9], CD133 [9], and SH3GLB1 [12]. SOD2 is associated with CD133 expression derived from TMZ-induced ROS [9] and mitochondrial ROS is related to stem cell homeostasis [20]. Therefore, ROS might increase SH3GLB1 levels in the same way as SOD2 and CD133. Furthermore, CD133^+^ cells with low SH3GLB1 levels are susceptible to cytotoxic drugs. However, the cells with high SH3GLB1 levels are not susceptible to cytotoxic drugs (Fig. 1). These results demonstrate that SH3GLB1 contributes to drug resistance in the TIC-featured cells and explain how different and complex classes of TICs exist in the cellular hierarchy.

In GBM, it has been known that CD133 is associated with cell proliferation, self-renewal, and resistance against TMZ [21]. In contrast, CD133 expression can be regulated by stress factors, cell signaling, epigenetic alterations, and microRNAs [22]. We further found that SH3GLB1 can affect CD133 transcription through changing histone H4K5 acetylation at its promoter (Figs. 3, 4 and 5). Given that AcH4K5 is related to class I histone deacetylases (HDAC) inhibitor [23] and suberoylanilide hydroxamine (SAHA), a pan-HDAC inhibitor, suppressed the class I HDACs and class II HDACs, including HDAC1, HDAC2, HDAC3 and HDAC6 [24]. We found that SAHA simultaneously increased levels of SH3GLB1, AcH4K5 and CD133 in parental U87MG-CD133^+^ enriched cells (High CD133 levels and low SH3GLB1 levels) (Fig. S2B). It implies that there is some HDAC enzyme reversely involved in up-regulation of SH3GLB1 expression, which further affects levels of AcH4K5 and CD133. Accordingly, CD133^+^ gliomaspheres depend on OXPHOS [25], mitochondrial metabolites can be co-factors for epigenetic modification [26], and mitochondrial activity influences H4K5 acylation [14]. In addition, SH3GLB1 can control mitochondrial membrane potential and ATP generation following TMZ treatment [12]. These studies supporting our findings demonstrate that SH3GLB1, a mitochondrial metabolism-related factor, can contribute to epigenetic changes in CD133 gene expression related to TIC features and acquired resistance.

## Conclusions

**Fig. 6:**
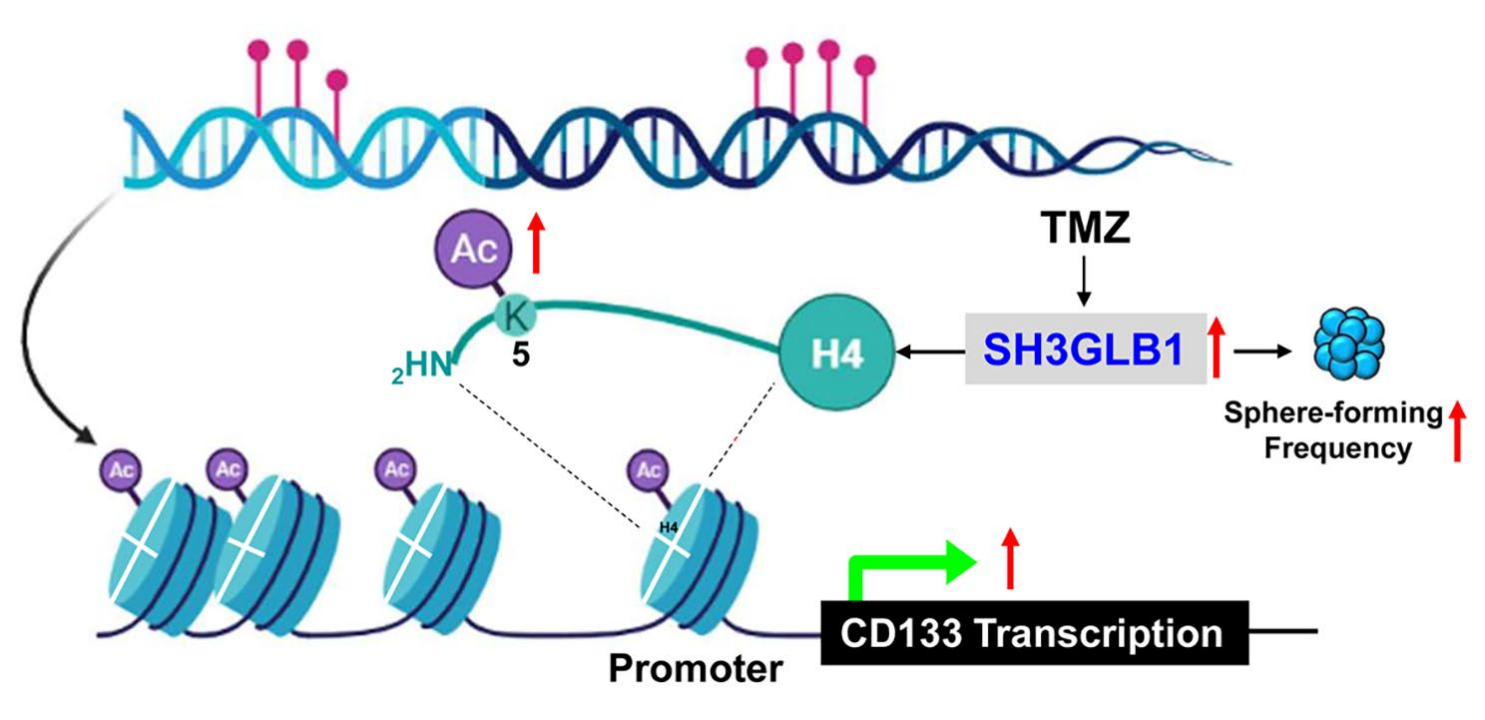
The schematic diagram illustrates the role of SH3GLB1 on spheroid formation and regulation of CD133 transcription in GBM cells. GBM: Glioblastoma

In this study, we showed that SH3GLB1 could affect spheroid formation and regulate CD133 expression through H4K5 acetylation of the promoter (Fig. 6). The findings show that the GBM-resistant cells with high SH3GLB1 levels can contribute to enhanced TIC-features, such as genes and morphology, leading to an increase in cell resistance to drug treatment.

## Electronic supplementary material

Below is the link to the electronic supplementary material.

Supplementary Material 1

## Data Availability

All data and materials in the present study are available from the corresponding author upon reasonable request.

## Abbreviations

Bif-1: Bax interacting factor 1
ChIP: Chromatin immunoprecipitation
ECL: Enhanced chemiluminescence
ELDA: Extreme Limiting Dilution Analysis
GBM: Glioblastoma
H4K5 acetylation: Histone 4 lysine 5 acetylation
H_2_O_2_: Hydrogen peroxide
MGMT: O^6^-methylguanine-DNA methyltransferase
PVDF: Polyvinylidene difluoride
SDS-PAGE: Sodium dodecyl sulfate-polyacrylamide gel electrophoresis
shRNA: Short hairpin RNA
siRNA: Small interfering RNA
SOD2: Superoxide dismutase 2
TICs: Tumor-initiating cells
TMZ: Temozolomide
TMZ-R: Temozolomide resistance
