# Supplementary material for: Role of SH3GLB1 in the regulation of CD133 expression in GBM cells
Source: BMC Cancer. 2023 Jul 31;23:713. doi: 10.1186/s12885-023-11211-8 (PMC10391956; doi:10.1186/s12885-023-11211-8)
Supplement: Supplementary file 1 — Supplementary Material 1 [file 12885_2023_11211_MOESM1_ESM.pdf]

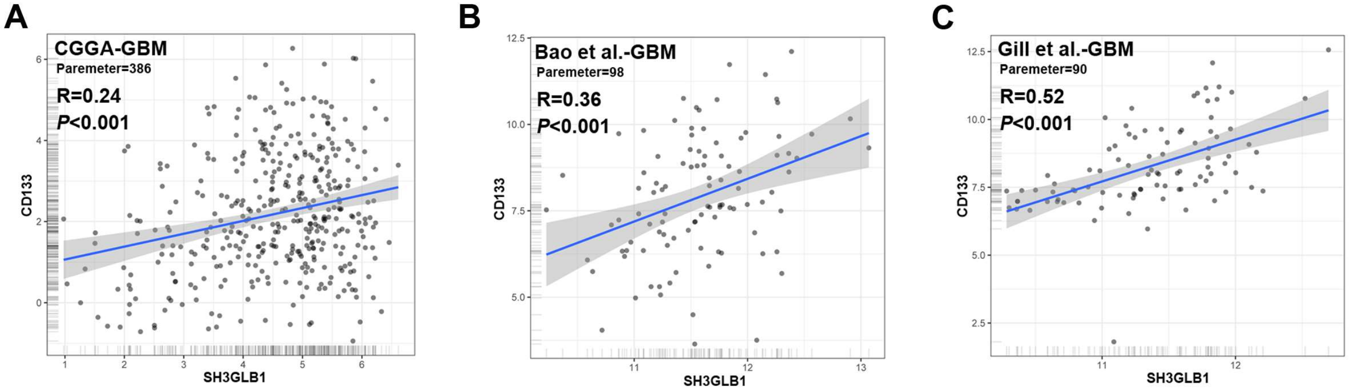

Fig. S1 Gene-expression levels of SH3GLB1 and CD133 from the GBM databases of (A) Chinese Glioma Genome Atlas (CGGA), (B) Bao et al. [1] and (C) Gill et al. [2] are shown in scatter plots with correlation assessment.

[1] Bao ZS, Chen HM, Yang MY, Zhang CB, Yu K, Ye WL, et al. RNA-seq of 272 gliomas revealed a novel, recurrent PTPRZ1-MET fusion transcript in secondary glioblastomas. Genome Res. 2014;24(11):1765-73.

[2] Gill BJ, Pisapia DJ, Malone HR, Goldstein H, Lei L, Sonabend A, et al. MRI-localized biopsies reveal subtype-specific differences in molecular and cellular composition at the margins of glioblastoma. Proc Natl Acad Sci U S A. 2014;111(34):12550-5.

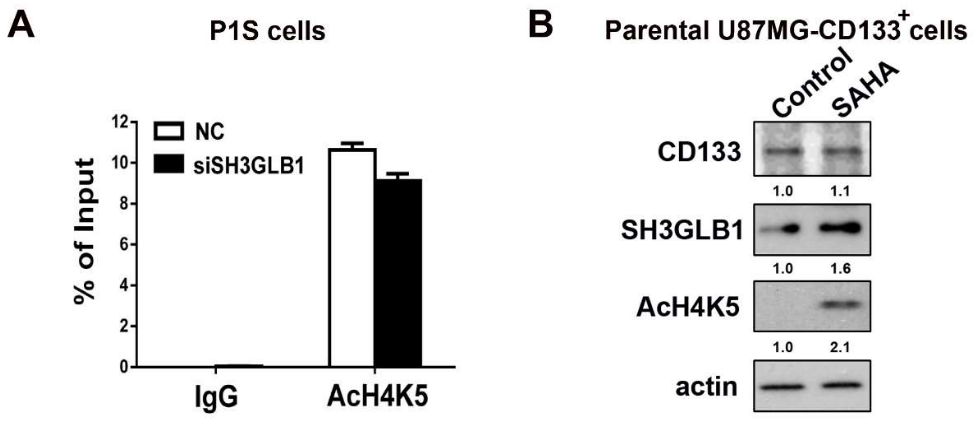

Fig. S2 (A) Reduced histone H4K5 acetylation to the CD133 promoter following SH3GLB1 knockdown is shown by chromatin immunoprecipitation assay in the primary P1S resistant cells. (B) The protein levels of CD133, SH3GLB1 and AcH4K5 were shown in the parental U87MG-CD133^{+} cells after suberoylanilide hydroxamine (SAHA; 10μM) treatment for 24 hours. All the blots were cropped prior to hybridization with primary antibodies. The original blots are presented in Fig. S6.

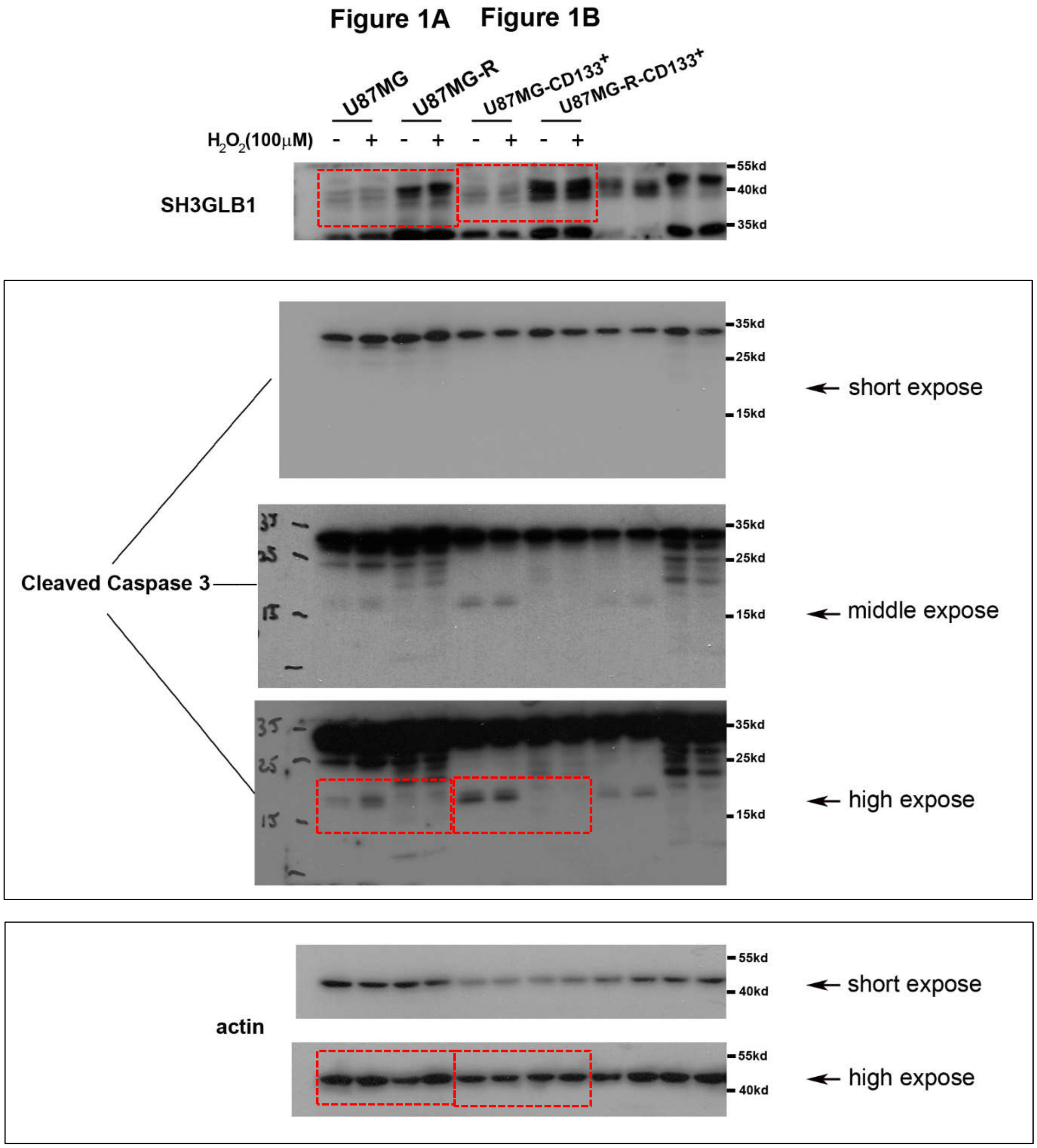

Fig. S3 The original images of western blotting in Figure 1 A and 1B. Since the positions where the protein blots appeared were quite stable and for obtaining clearer bands, we set the upper and lower boundaries of the membranes according to protein molecular weight, and the left and right boundaries were according to different cell lines or other experiments. Therefore, all the blots were cropped prior to hybridization with

primary antibodies. The red dashed boxes in the original blots indicate edges of membrane in Figure 1 A and 1B of the manuscript.

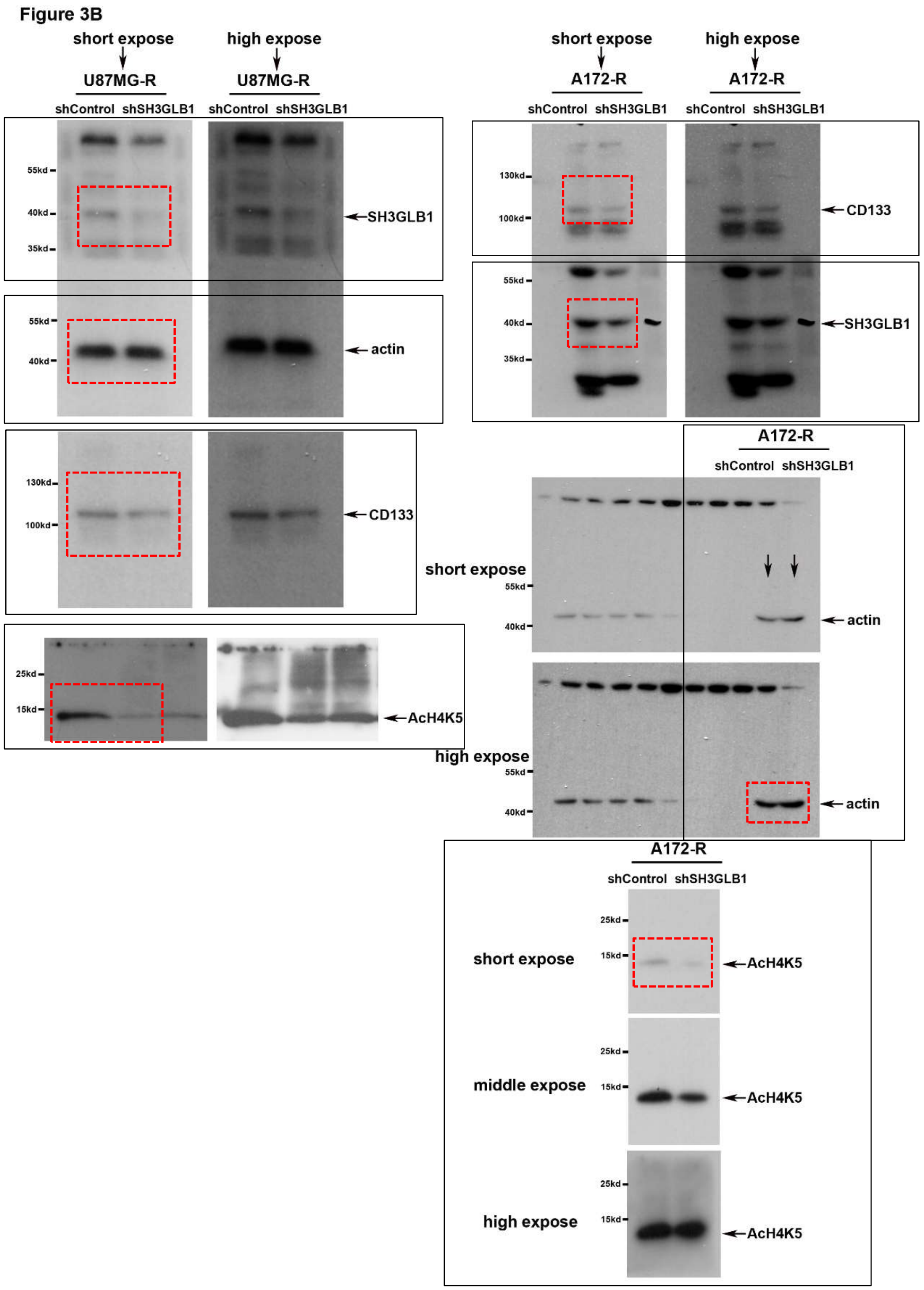

Fig. S4 The original images of western blotting in Figure 3B. Since the positions where the protein blots appeared were quite stable and for obtaining clearer bands, we set the upper and lower boundaries of the membranes according to protein molecular weight,

and the left and right boundaries were according to different cell lines or other experiments. Therefore, all the blots were cropped prior to hybridization with primary antibodies. The red dashed boxes in the original blots indicate edges of membrane in Figure 3B of the manuscript.

Figure 3C

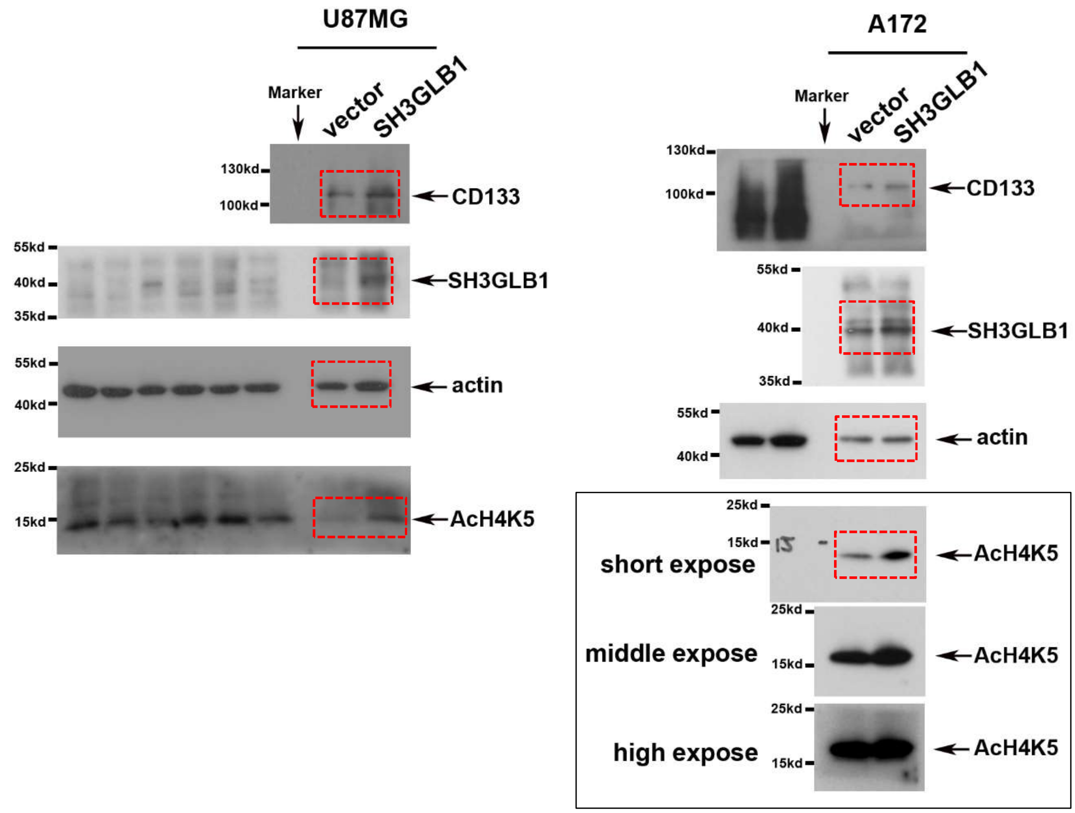

Fig. S5 The original images of western blotting in Figure 3C. Since the positions where the protein blots appeared were quite stable and for obtaining clearer bands, we set the upper and lower boundaries of the membranes according to protein molecular weight, and the left and right boundaries were according to different cell lines or other experiments. Therefore, all the blots were cropped prior to hybridization with primary antibodies. The red dashed boxes in the original blots indicate edges of membrane in Figure 3C of the manuscript.

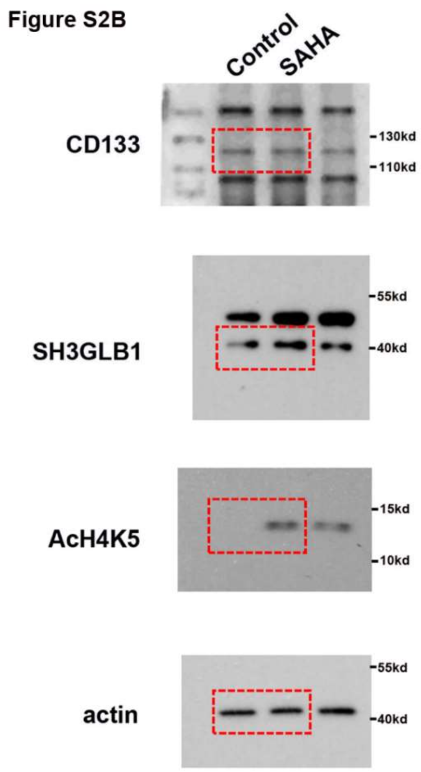

Fig. S6 The original images of western blotting in Figure S2B. Since the positions where the protein blots appeared were quite stable and for obtaining clearer bands, we set the upper and lower boundaries of the membranes according to protein molecular weight, and the left and right boundaries were according to different cell lines or other experiments. Therefore, all the blots were cropped prior to hybridization with primary antibodies. The red dashed boxes in the original blots indicate edges of membrane in Figure S2B.
